# Impact of sustainable feeds on omega-3 long-chain fatty acid levels in farmed Atlantic salmon, 2006–2015

**DOI:** 10.1038/srep21892

**Published:** 2016-02-22

**Authors:** M. Sprague, J.R. Dick, D.R. Tocher

**Affiliations:** 1 Institute of Aquaculture, School of Natural Sciences, University of Stirling, Stirling FK9 4LA, Scotland, UK

## Abstract

As the global population and its demand for seafood increases more of our fish will come from aquaculture. Farmed Atlantic salmon are a global commodity and, as an oily fish, contain a rich source of the health promoting long-chain omega-3 fatty acids, eicosapentaenoic (EPA) and docosahexaenoic (DHA) acids. Replacing the traditional finite marine ingredients, fishmeal and fish oil, in farmed salmon diets with sustainable alternatives of terrestrial origin, devoid of EPA and DHA, presents a significant challenge for the aquaculture industry. By comparing the fatty acid composition of over 3,000 Scottish Atlantic salmon farmed between 2006 and 2015, we find that terrestrial fatty acids have significantly increased alongside a decrease in EPA and DHA levels. Consequently, the nutritional value of the final product is compromised requiring double portion sizes, as compared to 2006, in order to satisfy recommended EPA + DHA intake levels endorsed by health advisory organisations. Nevertheless, farmed Scottish salmon still delivers more EPA + DHA than most other fish species and all terrestrial livestock. Our findings highlight the global shortfall of EPA and DHA and the implications this has for the human consumer and examines the potential of microalgae and genetically modified crops as future sources of these important fatty acids.

Omega-3 (n-3) fatty acids are widely recognised as being essential nutrients for the health and well-being of humans, particularly with respect to the n-3 long-chain polyunsaturated fatty acids (LC-PUFA), eicosapentaenoic (EPA; 20:5n-3) and docosahexaenoic (DHA; 22:6n-3) acids, that exert a range of health benefits through their molecular, cellular and physiological actions^1^. Fish are the major dietary source of n-3 LC-PUFA for humans as well as being an excellent source of protein, vitamin and minerals^2^. Recommendations for the suggested daily intake levels of EPA and DHA vary globally, depending on the regional scientific or health advisory organisations^3^. Nevertheless, most agree that the general population should be consuming at least two portions of fish per week, one of which should be oily^3^,^4^,^5^,^6^,^7^,^8^. However, with the global population and its demand for seafood increasing coupled with wild catch fisheries at, or beyond, exploitable limits, a greater proportion of fish destined for the table market are being farmed. Indeed, aquaculture has been the fastest growing animal-food producing sector for the past few decades, outpacing population growth, and currently supplies around 50% of the world’s fish and seafood for human consumption^9^.

Atlantic salmon (*Salmo salar* L.) represents an increasingly popular species in the global fish market, largely due to its high market value over low-value freshwater species. Today, salmon farming occurs throughout the world, with the main producers being Norway, Chile, Scotland and North America with a combined production volume of around 2 million metric tonnes^10^. Scottish salmon in particular is highly regarded throughout the world and has traditionally been marketed as a premium product, being the first fish and non-French food to receive the prestigious Label Rouge accolade in 1992 as well as being designated Protected Geographical Indication (PGI) by the European Commission in 2004^11^,^12^. Accordingly, farmed Scottish salmon was the UK’s most exported food product in 2014 generating an export value of around £500 million GBP (~$789M USD) with the USA, France and emerging markets in the Far East, namely China, being the main importers^13^. Despite the prospective role that aquaculture can play in feeding the ever-growing population, there is increasing concern over the impact that this fast-growing industry could have on wild fish stocks^14^,^15^,^16^,^17^,^18^,^19^.

As an oily fish, Atlantic salmon provide a high nutritional content to consumers, especially with respect to EPA and DHA. However, like humans, salmon along with other coldwater marine species of fish are inefficient at converting the shorter-chain fatty acid, α-linolenic acid (ALA; 18:3n-3), into EPA and DHA, and must therefore obtain the n-3 LC-PUFA through the diet^2^,^16^,^20^. Farmed salmon were traditionally fed a diet with high levels of the marine ingredients, fish oil and fishmeal, derived from pelagic fisheries. The continued pressures on wild fish stocks as aquaculture production grows along with greater competition for LC-PUFA sources from the nutraceutical and pharmaceutical industries have resulted in changes to aquaculture feed formulations. Terrestrial ingredients such as plant sources, mainly of oilseed origin, are increasingly used in salmon feeds without any detriment to fish health or growth^21^. Nevertheless, the fatty acid profiles of vegetable oils differ from those of fish oil, being richer in n-6 PUFA and devoid of n-3 LC-PUFA, resulting in changes to the fatty acid composition of farmed fish^16^,^21^,^22^,^23^. Consequently, the nutritional benefit to the final human consumer is lowered prompting the International Fish Meal and Fish Oil Organisation, the trade group representing the marine ingredients industry, to highlight their concerns over the declining levels of n-3 LC-PUFA in farmed salmon. Thus, we examined the changes in the fatty acid contents and compositions, particularly with respect to EPA and DHA, in the flesh of Scottish Atlantic salmon farmed between 2006 and 2015 and discuss the potential implications for the human consumer.

## Results and Discussion

### From marine- to plant-based feeds

**Figure 1:**
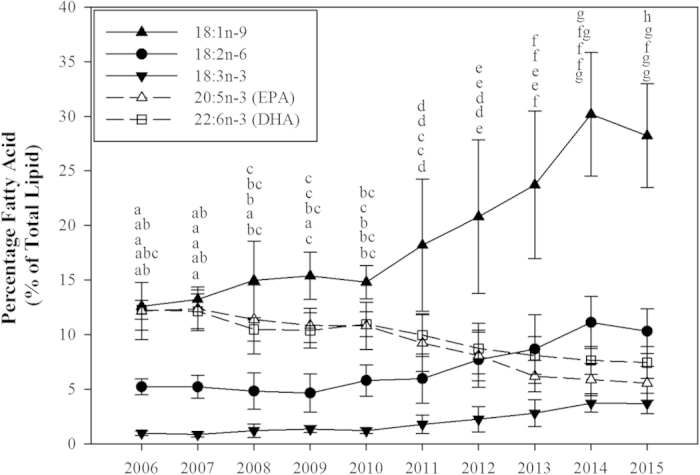
Changes in the levels of fatty acids (% of total fatty acids), of either marine (- - -) or terrestrial origin (^___^), in the flesh of Scottish Atlantic salmon farmed between 2006–2015 (mean ± SD). Stacked lettering above data points are in the order of 18:1n-9, EPA, DHA, 18:2n-6 and 18:3n-3 respectively, and indicate significant differences (*P* < 0.05) between years for the same fatty acid (n = 106, 174, 247, 81, 85, 393, 212, 523, 546 and 687 for 2006–2015 respectively).

The main nutritional issue for marine finfish aquaculture has been the sourcing of sustainable alternative ingredients to replace the finite and highly exploited marine resources, fish oil and fishmeal. The development of modern, sustainable feeds with increased use of terrestrial ingredients, mainly of oilseed origin, has had no major effect on salmon health or growth performance^21^ and has resulted in a concomitant decrease in the levels of undesirable contaminants (e.g. dioxins and PCBs) in salmon flesh^24^. Nonetheless, the change in dietary oil source presents a problem for the aquaculture industry as the fatty acid composition of fish muscle (flesh) reflects that of the diet^20^. Rapeseed oil is the most commonly utilised fish oil alternative in Europe, including Scotland, due to its favourable price and ready availability. However, while plant-based alternatives including rapeseed contain some types of n-3 fatty acids they lack the nutritionally beneficial n-3 LC-PUFA, EPA and DHA, found almost exclusively in fish oil and other marine sources (see Table 1). Consequently, the use of rapeseed oil in aquafeeds has resulted in increased levels of ‘terrestrial’ fatty acids such as oleic (18:1n-9), linoleic (18:2n-6) and, to a lesser extent, α-linolenic (18:3n-3). This trend is evident in the fatty acid profiles of farmed Scottish salmon and is particularly noticeable from 2010 onwards when the levels of ‘terrestrial’ fatty acids showed a greater rate of change, doubling from 15%, 5% and 2% in 2010 to ~30%, 10% and 5% in 2015 (18:1n-9, 18:2n-6 and 18:3n-3, respectively), while the marine fatty acids, EPA and DHA, fell by approximately half (Fig. 1 and Supplementary Table 1). The increased inclusion level of plant ingredients in salmon feeds accelerated with increased fish oil prices from 2009 brought about by the growing demand for this finite resource. This has led to similar changes in salmon compositions to that in Scotland being reported in both the Norwegian and Tasmanian salmon industries^25^,^26^, reflecting the changes in salmonid feed formulations throughout the years.

Although production of the global fed aquaculture industry increased from 15 to 35 million tonnes over the period 2000–2012, the level of fish oil used within the same period remained static at around 800,000 metric tonnes per year^7^,^27^,^28^. Aquaculture currently uses around 75% of the global fish oil supply with 21% going towards direct human consumption^27^,^28^. The increasing demand from aquaculture and the nutraceutical and pharmaceutical industries, coupled with natural climatic events such as El Niño affecting both fish oil supply and prices, resulted in the salmonid industry, as the biggest user of fish oil (~60%), necessarily reducing the overall levels of marine ingredients in feeds. For instance, fish oil inclusion levels in Norwegian salmon feeds fell from 24% in 1990 to 11% in 2013^25^. Consequently, commercial aquafeeds have increasingly incorporated blends of vegetable and fish or other oils to meet the nutritional requirements of the fish whilst also relieving pressure on marine resources, albeit with some compromise of the nutritional benefit to the human consumer.

### Potential consequences for the human consumer

The benefits of consuming n-3 LC-PUFA in the diet with respect to health and well-being are well known. Increasing the intake of EPA and DHA has been associated with a wide range of health-promoting roles such as in the development and function of neural and eye tissue, particularly in infants, as well as in reducing the risk of cardiovascular disease, inflammation, depression and other chronic conditions^1^,^29^,^30^,^31^,^32^. Farmed Atlantic salmon is often marketed for its health promoting properties, primarily its high n-3 LC-PUFA content. However, this benefit will decline as alternative ingredients, such as vegetable oils, replace fish oils in the feeds of salmon. For example, the greater use of vegetable ingredients in salmonid feeds has resulted in higher n-6 levels in farmed fish^21^,^22^,^23^,^26^, (see also Supplementary Table 1). Although n-6 fatty acids such as linoleic acid (18:2n-6) are considered essential in the diet, there is already a very high intake in the human diet such that an imbalance of n-6/n-3 may affect the uptake and function of the more beneficial n-3 LC-PUFA^1^,^33^.

**Figure 2:**
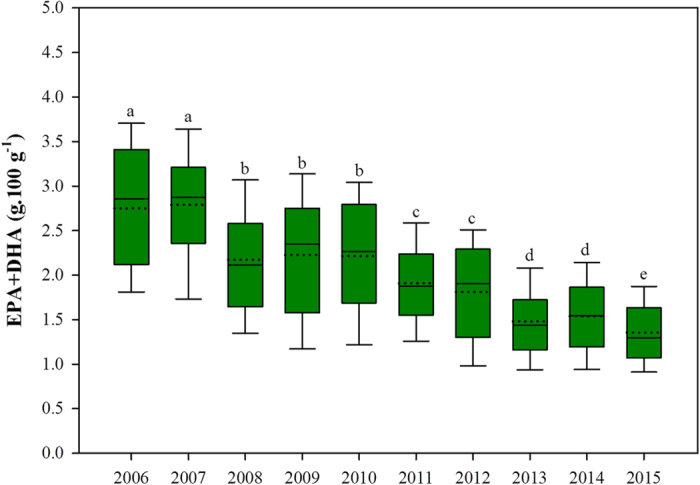
Levels of EPA + DHA (g.100 g^−1^) in farmed Scottish Atlantic salmon between 2006 and 2015. Median (—), mean (^…^), interquartile range (box) and 10^th^ and 90^th^ percentiles (whiskers) are presented. Significant differences (*P* < 0.05) between mean values are indicated by different lettering (n = 106, 174, 247, 81, 85, 393, 212, 523, 546 and 687 for 2006–2015 respectively).

Absolute contents of EPA and DHA in the flesh of farmed Scottish Atlantic salmon have fallen significantly in recent years, decreasing on average from 2.74 g in 2006 to 1.36 g per 100 g wet weight (ww) serving in 2015 (Fig. 2). Nonetheless, this is still comparatively higher than the 1.02 g, 1.36 g and 1.00 g EPA + DHA per 100 g ww serving reported in Norwegian farmed salmon in 2011^34^, 2012^25^ and 2013^35^ respectively. Although both the Scottish and Norwegian salmon farming industries are served by the same three main commercial feed producers, differences in feed formulations between countries exist. The Norwegian salmon industry for example appears to focus largely on sustainability and produces a narrow range of product specifications, all with decreased reliance on marine ingredients in favour of terrestrial alternatives^25^. In contrast, the Scottish salmon industry is more complex and bespoke catering for both the value and premium markets primarily driven by retailers, the former being variable in n-3 LC-PUFA content and the latter formulated to deliver a high level of n-3 LC-PUFA such that one portion provides an adequate weekly intake for humans. This has resulted in a wide range of feeds being produced with formulation data difficult to access due to retailer confidentiality. This is clearly evident from the variation and ranges of the fatty acid composition and content data presented in Figs 1 and 2.

**Figure 3:**
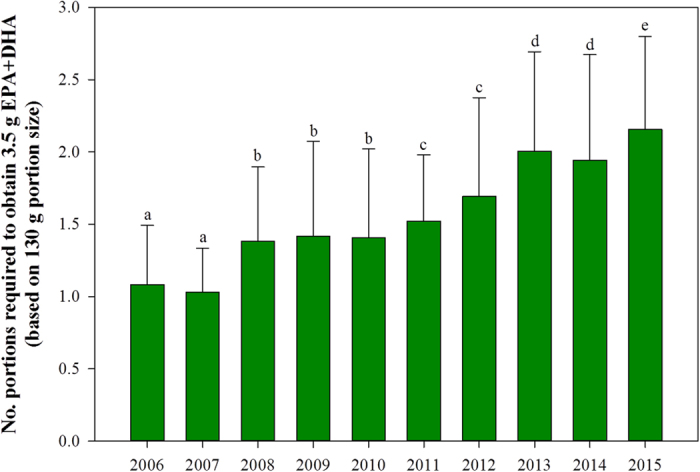
Number of portions required (mean ± SD) to obtain a weekly intake of 3.5 g EPA + DHA (ISSFAL, 2004)^36^, based on a 130 g serving^37^. Significant differences (*P* < 0.05) are indicated by different lettering (n = 106, 174, 247, 81, 85, 393, 212, 523, 546 and 687 for 2006–2015 respectively).

Public health bodies such as the UK’s Food Standards Agency (FSA) and the American Heart Association (AHA) currently advise consuming at least two portions of fish per week, of which one should be oily^3^,^4^,^5^,^6^,^7^,^8^. With EPA and DHA levels in farmed salmon decreasing, a more effective approach would be through the establishment of Dietary Reference Intake (DRI) levels. However, there is no general consensus among health and scientific organisations, with 51 National and International expert bodies all suggesting variable DRI levels according to an individual’s health status^3^. For example, the AHA has no recommended DRI levels for the ordinary consumer but suggests a daily intake of 1.0 g EPA + DHA for those individuals with established cardiovascular disease^7^,^8^. The European Food Safety Authority (EFSA) suggest that all adults should be consuming 250 mg EPA + DHA per day respectively through oily fish consumption^36^, whereas the International Society for the Study of Fatty Acids and Lipids (ISSFAL) recommends a minimum daily intake of 500 mg to reduce the risk of cardiovascular disease^37^. Based on a 130 g portion, as advised by EFSA^38^, one portion of farmed Scottish salmon in 2015 would more than satisfy the daily DRI levels mentioned above, providing 1.8 g EPA + DHA. Nevertheless, since fish and seafood are the major dietary source of EPA and DHA in the human diet and are generally limited to one to two meals per week, it may be more appropriate to express the DRI on a weekly basis. Thus, a single 130 g portion of Scottish salmon farmed in 2006 would have been adequate to meet the 3.5 g EPA + DHA weekly intake level set by ISSFAL, whereas in 2015 this would have required two portions (Fig. 3). However, it is important to highlight that while the present results relate to Scottish averages there will be salmon products which will require both larger or smaller portion sizes to meet the DRI, based on the levels of fish oil in their diets. Few retailers state the EPA and DHA content on pre-packaged salmon products at present^35^, possibly due to the changing levels of fish oils in diets but also due to inter-pack variation as both lipid and fatty acid content can vary according to the type of cut consumed^39^,^40^.

### Farmed versus wild

Aquaculture is often viewed in a less favourable perspective than some other farming sectors. One popular misconception among consumers is that farmed fish are inferior in terms of quality and nutritional content than wild fish. In reality, farmed fish have been found to contain as much or, in most instances, more grams of EPA + DHA per serving than their wild-caught counterparts^6^,^35^,^38^. Moreover, it is often quoted that farmed salmon contains more fat and is less healthy than wild salmon. Lipids (fat) supply approximately twice the amount of energy as either protein or carbohydrates. Farmed fish such as Atlantic salmon are often fed a high-energy (lipid) diet to ensure optimal growth rate while sparing the more expensive dietary protein for conversion into muscle protein^20^. The lipid content of Scottish Atlantic salmon farmed between 2006 and 2015 remained largely consistent (~12–13%), although some significant differences were observed between years (Supplementary Table 1). Nonetheless, these values are within the range reported elsewhere for farmed Atlantic salmon from different regions^25^,^26^, as well as for farmed salmon products found in UK supermarkets^35^. Wild salmon are capable of accumulating similar lipid levels to farmed salmon^41^. However, the lipid content of wild fish varies according to its nutritional state, which is affected by season, food availability, species, age, sex and developmental or reproductive status^20^,^42^. The lower levels (~4%) generally reported for wild salmon relate to the time when they are caught, typically during their return migration to rivers to spawn when the large lipid deposits accumulated have been mobilised and depleted to support gonadal development as well as supply the energy expended during migration itself^42^.

**Figure 4:**
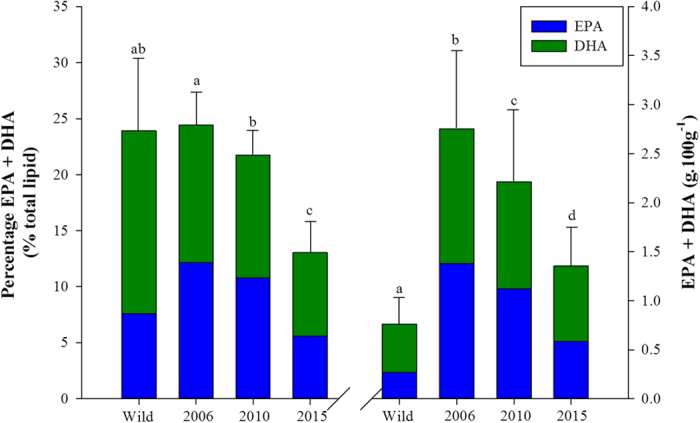
Differences in the proportions (% of total fatty acids) and absolute levels (g.100 g^−1^) of EPA + DHA fatty acids between wild (Pacific species) and farmed Scottish Atlantic salmon from 2006, 2010 and 2014 (mean ± SD). Bars bearing different lettering indicate significant differences (*P* < 0.05) (n = 21 for wild salmon and 106, 85 and 687 for Scottish Atlantic salmon farmed in 2006, 2010 and 2015 respectively). Stacked bars represent contribution of EPA and DHA to total values for comparative purposes.

**Figure 5:**
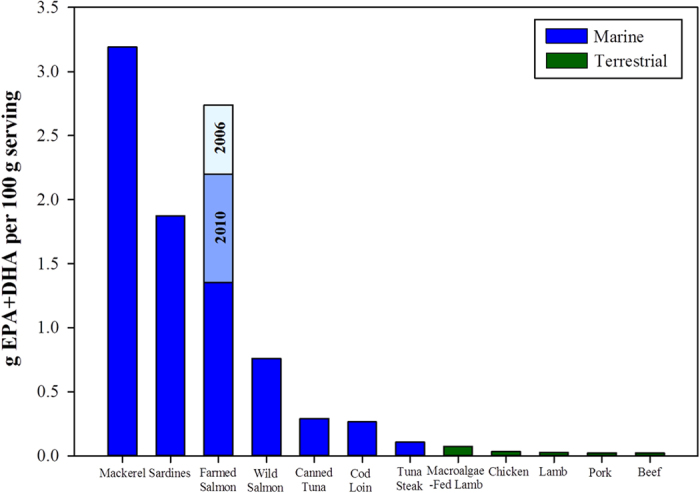
Comparison between the levels of EPA + DHA (g.100 g^−1^) in Scottish farmed Atlantic salmon compared to other fish species (blue) and terrestrially (green) produced animals. Stacked bars for farmed Scottish Atlantic Salmon indicate the decline in EPA + DHA levels for 2006, 2010 and 2015 respectively. All samples are based on duplicate analysis and analysed in 2014/15, with exception to wild salmon (Pacific species, n = 21) and Scottish farmed Atlantic salmon (n = 106, 85 and 687 for 2006, 2010 and 2015 respectively). Refer to methodology for further information on species sampled.

The aforementioned difference in lipid content between wild and farmed fish greatly affects the nutritional content, although the overall n-3 LC-PUFA levels in the flesh are ultimately determined by the levels in the feed. Fatty acid profiles are often presented as a percentage of the total lipid. This often leads to a further misinterpretation made by consumers regarding the perceived higher n-3 LC-PUFA nutritional content of wild fish compared to their farmed equivalents. In 2006, farmed Scottish Atlantic salmon flesh contained a similar proportion of EPA and DHA in the total lipid of their flesh to wild salmon (~24%, Fig. 4), owing to a high inclusion level of fish oil in the diet resulting in a fatty acid profile similar to that of the natural diet. Conversely, by 2010 the proportion of EPA and DHA in flesh lipid had significantly fallen to 21.7% and by 2015 to 13.0% which, at first glance, would suggest that wild salmon are more nutritionally beneficial to the human consumer. However, when expressed in absolute terms, *i.e.* grams of EPA + DHA per 100 g ww serving, which takes into account the lipid content of the flesh we find that, despite the reduction in EPA and DHA levels due to the increased use of plant ingredients in salmon feeds, farmed Atlantic salmon still provide a significantly higher amount of EPA + DHA than wild salmon, yielding 2.75, 2.21 and 1.36 g per 100 g ww flesh for salmon farmed in 2006, 2010 and 2015 respectively, compared to 0.76 g per 100 g ww flesh for wild salmon (Fig. 4). Furthermore, despite the decrease in the levels, farmed Scottish salmon still delivers more EPA + DHA than most other fish species as well as all farmed terrestrial livestock (Fig. 5). Nevertheless, if the continuing trend for the increased use of plant ingredients in salmon feeds endures then the risk of negating the beneficial effects from farmed salmon consumption will be realised.

### Future sources of EPA and DHA

Fish oil is still the main dietary source of EPA and DHA in the human diet. It is estimated that the annual demand for n-3 LC-PUFA to supply the global population of seven billion with 500 mg EPA + DHA per day, as recommended by ISSFAL^37^, is 1.25 million metric tonnes which is far greater than the estimated current total supply from all sources of just over 0.8 million metric tonnes^2^,^43^. Therefore, there is a major chronic shortfall in EPA and DHA to supply human requirements and demand. The aquaculture industry has employed several strategies to reduce fish oil use while maintaining the nutritional benefits of farmed salmon consumption. This not only includes the blending of fish and vegetable oils in commercial feeds as previously mentioned but also extends to the use of finisher feeds, whereby fish are fed a plant-based diet for the major duration of the ongrowing production cycle before switching to a fish-oil diet in the final weeks prior to harvest to increase the content of beneficial n-3 LC-PUFA^21^,^22^,^23^. However, this method still relies upon the inclusion of fish oil in the feeds.

In the marine environment, microalgae are the primary producers of n-3 LC-PUFA that are subsequently consumed and accumulated through the food chain to give the high levels of EPA and DHA that we generally find in marine, especially oily, fish. Therefore, microalgae represent potential feedstock for food and feed. Indeed, algal products are already used in human nutrition, particularly in the infant formula market, with 75% of the production volume used in the health food market as dietary supplements^44^. Accordingly, microalgae have been investigated as a promising alternative to the traditional marine derived ingredients in fish feeds, although these have largely been limited to DHA-producing species^45^,^46^. The main production of n-3 LC-PUFA by microalgae has predominantly focussed on heterotrophic species (e.g. *Schizochytrium*, *Crypthecodinium* and *Ulkena* species) that are cultivated by fermentation similar to yeasts under controlled conditions that give a higher production efficiency^44^. However, this technology is still regarded as being in the development stage resulting in low production volumes and higher costs^44^ which, at present, are far more expensive than for fish oil and meal^45^. Nevertheless, the main issues concerning the industry with respect to increasing the scale of production while simultaneously decreasing the production cost are expected to be addressed within the next few years^44^. Thus, in the future, microalgae will likely become available as a feed ingredient although, at present, they are unlikely to fulfil aquaculture demands for n-3 LC-PUFA. Further alternative marine sources of n-3 LC-PUFA include the use of krill^47^ or calanoid copepods^48^, although again production volumes are expected to be low and there is concern over the possible effects that harvesting down the trophic chain may have on higher trophic species that are normally reliant on these zooplankton^49^.

Perhaps surprisingly, future *de novo* n-3 LC-PUFA sources may originate from land-based supplies. Genetically modified (GM) oilseed crops have been engineered to synthesise the n-3 LC-PUFA, EPA and DHA, not normally associated with plant sources. This involves inserting specific microalgae genes that encode for the biosynthetic enzymes required to produce these specific fatty acids to generate seeds that accumulate oils with up to 20% of total fatty acids as n-3 LC-PUFA^50^,^51^. These GM crops have been demonstrated to be an effective replacement for fish oil in salmon feeds^43^,^52^. Although there is currently no commercial production, the potential for production to increase rapidly means that in the near future volumes could be considerable, although changing public perceptions and attitudes towards GM products are required, particularly in some European countries, before these GM oils can be used on a commercial scale. Nonetheless, an n-3 LC-PUFA GM yeast strain is currently being used within the Chilean salmon industry to give a niche product^53^ although, as with microalgae production, the fermentation technology used to produce the yeast is currently expensive and unlikely to produce sufficient volumes to meet aquaculture demand in the near-future. However, both the microalgae and GM technologies offer clear potential to counter the global deficit of n-3 LC-PUFA sources and reverse the trend of decreasing levels of EPA and DHA in farmed fish and thus ensure that farmed Atlantic salmon will continue to maintain its nutritional benefit to the human consumer in the future.

## Materials and Methods

### Sample data

The present study examined over 3,000 fatty acid profiles of farmed Scottish salmon analysed between 2006 and 2015 by the Nutrition Analytical Service (NAS), Institute of Aquaculture, University of Stirling. Samples were provided year-round by a wide range of salmon producers and feed manufacturers, reflecting the continual production of the Scottish salmon industry. All samples included in the study were deemed to be representative of the commercial Scottish salmon industry, with trial fish, fish purchased from supermarkets or whose farmed location was otherwise unknown excluded from the study. The samples were processed by NAS in a laboratory working towards ISO/IEC 17025 accreditation. Briefly, samples of standard quality cuts, a standardised muscle section used in flesh quality determination corresponding to the region of flesh between dorsal fin and anal fin, were shipped to NAS on ice and processed that day. All samples were skinned and boned before being thoroughly homogenised prior to analysis.

To compare the EPA and DHA levels (g.100 g^−1^ ww) in farmed Scottish salmon with other marine and terrestrial food products, samples of fresh Atlantic mackerel (*Scomber scombrus*) fillets, Atlantic cod (*Gadus morhua*) loin, tuna steak (Yellowfin tuna, *Thunnus albacares*), beef, chicken breast, lamb, macroalgae-fed lamb (North Ronaldsay, Orkney, Scotland) and pork meat, together with canned tuna in spring water (Skipjack tuna, *Katsuwonus pelamis*) and tinned sardines in spring water (*Sardina pilchardus*) were purchased from local retailers during late 2014/early 2015 and analysed as previously described. Wild salmon samples (*n* = 21), generally Pacific salmon species (*Oncorhynchus kisutch*, *O. nerka* or *O. keta*), were purchased from supermarkets between 2006 and 2015 and analysed as described for farmed salmon. No differences in EPA + DHA content (g.100 g^−1^ ww) were observed between years, thus wild salmon samples were pooled.

### Lipid and fatty acid determination

Total lipid was extracted from ~0.5 g of homogenised salmon flesh in 20 volumes of ice-cold chloroform/methanol (2:1 v/v) using an Ultra-Turrax tissue disruptor (Fisher Scientific, Loughborough, UK) and the lipid determined gravimetrically^54^. Fatty acid methyl esters (FAME) from total lipid were prepared by acid-catalysed transmethylation at 50 °C for 16 h^55^. FAME were extracted and purified as described previously^56^, and separated and quantified by gas-liquid chromatography using a Fisons GC-8160 (Thermo Scientific, Milan, Italy) equipped with a 30 m × 0.32 mm i.d. × 0.25 μm ZB-wax column (Phenomenex, Cheshire, UK), ‘on column’ injection and flame ionisation detection. Hydrogen was used as carrier gas with an initial oven thermal gradient from 50 °C to 150 °C at 40 °C.min^−1^ to a final temperature of 230 °C at 2 °C.min^−1^. Individual FAME were identified by comparison to known standards (Supelco™ 37-FAME mix; Sigma-Aldrich Ltd., Poole, UK) and published data^56^. Data were collected and processed using Chromcard for Windows (Version 1.19; Thermoquest Italia S.p.A., Milan, Italy). Fatty acid content per g of tissue was calculated using heptadecanoic acid (17:0) as internal standard.

### Statistical analysis

Statistical analyses were performed using Minitab^®^ v.17.1.0 statistical software (Minitab Inc., PA, USA). Data were assessed for normality with Kolmogorov-Smirnov test and for homogeneity of variances by Bartlett’s test and examination of residual plots and, where necessary, transformed using arcsine or natural logarithm transformation. Data were compared by a one-way analysis of variance (ANOVA) with replicate post hoc comparisons made using Tukey’s test^57^. A significance of *P* < 0.05 was applied to all statistical tests performed. Results are presented as mean ± standard deviation unless otherwise stated.

## Additional Information

**How to cite this article**: Sprague, M. *et al.* Impact of sustainable feeds on omega-3 long-chain fatty acid levels in farmed Atlantic salmon, 2006-2015. *Sci. Rep.* **6**, 21892; doi: 10.1038/srep21892 (2016).

## Supplementary Material

Supplementary Table 1

## Figures and Tables

**Table 1:** Examples of differences in fatty acid compositions of various oil sources of either terrestrial or marine origin.

	Terrestrial						Marine			
	Rapeseed	Soyabean	Sunflower	Linseed	Borage	Camelina	Fish Oil^a^	Fish Oil^b^	Algal Oil^c^	Algal Oil^d^
*Fatty acid (% of total lipid*)										
14:0	0.1	0.1	0.1	0.1	0.1	0.1	7.5	7.1	3.6	1.1
16:0	4.7	11.4	6.1	5.2	11.0	5.1	18.0	13.8	37.9	16.1
18:0	1.9	4.0	3.5	3.5	3.7	2.6	3.6	1.5	1.1	1.9
20:0	0.6	0.4	0.3	0.1	0.2	1.6	0.2	0.2	0.1	0.4
*Total saturates*^1^	*7.7*	*16.5*	*10.7*	*9.1*	*15.1*	*9.9*	*29.9*	*23.6*	*44.1*	*20.2*
16:1n-7	0.2	0.1	0.1	0.1	0.2	0.1	8.9	5.9	0.2	0.1
18:1n-9	62.4	27.4	28.2	18.7	16.1	17.4	7.7	9.7	0.5	26.9
18:1n-7	3.1	1.8	0.8	0.7	0.7	1.1	3.1	2.0	0.3	0.4
20:1n-9	1.3	0.3	0.2	0.2	4.0	14.2	1.1	11.6	–	–
22:1n-11	–	–	0.1	–	–	–	0.9	17.3	–	–
22:1n-9	0.1	–	–	–	2.5	2.6	0.1	1.2-	–	–
24:1n-9	0.4	–	–	–	1.6	0.5	0.4	0.8	0.3	0.4
*Total monoenes*^2^	*67.5*	*29.6*	*29.5*	*19.8*	*25.1*	*36.4*	*22.8*	*49.0*	*1.3*	*28.0*
18:2n-6	16.4	48.9	59.4	15.6	37.7	19.3	1.2	1.4	0.3	1.9
18:3n-6	–	–	–	–	21.7	–	0.3	0.2	0.1	–
20:2n-6	0.1	–	–	–	0.2	1.5	0.1	0.2	–	–
20:4n-6	–	–	–	–	–	0.1	1.0	0.4	0.8	1.1
22:5n-6	–	–	–	–	–	–	0.4	0.1	7.4	1.0
*Total n-6 PUFA*^3^	*16.5*	*48.9*	*59.4*	*15.6*	*59.5*	*20.9*	*3.3*	*2.4*	*8.7*	*4.2*
18:3n-3	8.3	5.1	0.3	55.5	0.2	31.9	0.9	1.1	0.2	0.1
18:4n-3	–	–	–	–	0.1	–	3.2	3.4	0.3	0.1
20:3n-3	–	–	–	0.1	–	1.0	0.1	0.1	–	0.1
20:5n-3 (EPA)	–	–	–	–	–	–	18.8	8.4	0.8	16.5
22:5n-3	–	–	–	–	–	–	2.2	0.9	0.1	3.4
22:6n-3 (DHA)	–	–	–	–	–	–	12.4	8.7	44.0	27.1
*Total n-3 PUFA*^4^	*8.3*	*5.1*	*0.3*	*55.5*	*0.3*	*32.9*	*38.2*	*23.5*	*45.9*	*47.7*
*Total PUFA*^5^	*24.8*	*54.0*	*59.7*	*71.1*	*59.8*	*53.8*	*47.3*	*27.4*	*54.6*	*51.8*
*n-3:n-6*	*0.5*	*0.1*	*<0.1*	*3.6*	*<0.1*	*1.6*	*11.6*	*9.8*	*5.3*	*11.4*

^1^Includes 15:0, 22:0 and 24:0.

^2^Includes 16:1n-9, 17:1, 20:1n-11 and 20:1n-7.

^3^Includes 20:3n-6 and 22:4n-6.

^4^Includes 20:4n-3.

^5^Includes 16:2, 16:3 and 16:4.

^a^South American fish oil (e.g. anchovy).

^b^Northern hemisphere fish oil (e.g. herring).

^c^DHA-rich *Schizochytrium* sp. algal oil.

^d^EPA + DHA *Schizochytrium* sp. algal oil (DSM nutritional products).
