# Supplementary material for: Impact of sustainable feeds on omega-3 long-chain fatty acid levels in farmed Atlantic salmon, 2006–2015
Source: Sci Rep. 2016 Feb 22;6:21892. doi: 10.1038/srep21892 (PMC4761991; doi:10.1038/srep21892)
Supplement: Supplementary Table 1 [file srep21892-s1.pdf]

Impact of sustainable feeds on omega-3 long-chain fatty acid levels in farmed Atlantic salmon, 2006-2015

Sprague, M.*, Dick, J.R. and Tocher, D.R.

Institute of Aquaculture, School of Natural Sciences, University of Stirling, Stirling FK9 4LA, Scotland, UK.

Supplementary Table 1. Fatty acid composition of Scottish Atlantic salmon (*Salmo salar*) farmed between 2006 and 2015. Means (±SD) bearing different superscript lettering within same row denote statistical differences (P<0.05).

	Sample Year									
	**2006**	**2007**	**2008**	**2009**	**2010**	**2011**	**2012**	**2013**	**2014**	**2015**
n	*106*	*174*	*247*	*81*	*85*	*393*	*212*	*523*	*546*	*687*
**Lipid (%)**	13.3±3.4[abc]	13.5±3.2[a]	11.8±3.1[d]	12.4±4.3[abcde]	11.9±3.5[bcde]	12.1±3.3[de]	12.7±2.6[abce]	12.3±3.6[cde]	13.2±3.3[a]	12.1±2.6[de]
Fatty acid content (% of total lipid)										
14:0	5.6±0.5[a]	5.4±0.7[ab]	5.3±0.9[b]	5.7±0.7[a]	5.7±0.4[a]	5.2±0.9[b]	5.2±1.1[b]	4.6±1.0[c]	3.5±0.8[d]	3.7±0.7[e]
16:0	16.1±0.8[a]	16.3±1.2[a]	15.1±1.6[b]	16.1±1.5[a]	16.1±1.1[a]	15.1±2.0[b]	15.1±1.8[b]	13.4±1.6[c]	12.3±1.4[d]	12.5±1.4[d]
18:0	3.6±0.4[a]	3.8±0.5[b]	3.3±0.8[c]	3.5±0.5[a]	3.5±0.5[a]	3.2±0.5[c]	3.5±0.5[a]	3.1±0.3[d]	3.0±0.2[e]	2.9±0.3[f]
20:0	0.1±0.0[a]	0.2±0.0[b]	0.2±0.1[b]	0.2±0.0[b]	0.2±0.0[b]	0.2±0.0[c]	0.3±0.1[d]	0.3±0.1[d]	0.3±0.0[e]	0.3±0.0[e]
Total Saturated[1]	***25.9±1.4[a]***	***26.2±2.2[a]***	***24.3±2.8[b]***	***26.0±2.6[a]***	***26.0±1.7[a]***	***24.2±3.1[b]***	***24.6±3.2[b]***	***21.8±2.7[c]***	***19.5±2.4[d]***	***19.7±2.3[d]***
16:1n-7	7.1±0.4[abc]	7.2±0.6[ab]	6.9±1.2[bc]	7.6±1.2[a]	7.2±0.7[abc]	7.0±1.5[bc]	6.8±1.5[c]	5.4±1.2[d]	4.3±1.2[e]	4.5±1.0[f]
18:1n-9	12.6±2.2[a]	13.2±0.9[ab]	15.0±3.6[c]	15.5±1.8[c]	14.8±1.5[bc]	18.2±6.1[d]	20.8±7.0[e]	23.7±6.8[f]	30.2±5.7[g]	28.2±4.8[h]
18:1n-7	3.3±0.2[abcd]	3.3±0.3[abe]	3.2±0.5[d]	3.4±0.3[ef]	3.3±0.4[abcde]	3.3±0.4[bc]	3.5±0.5[f]	3.3±0.3[abe]	3.4±0.4[ae]	3.2±0.2[de]
20:1n-9	3.6±1.8[abc]	2.8±2.1[d]	4.6±2.8[a]	3.7±2.0[abc]	3.3±2.3[bcd]	3.9±2.2[ab]	3.3±1.8[cd]	5.0±2.4[e]	3.1±1.7[cd]	3.9±1.4[a]
22:1n-11	3.6±1.9[abc]	2.8±2.3[d]	4.7±3.4[ab]	3.4±2.6[bcd]	3.5±3.3[cd]	4.2±3.1[abc]	2.8±2.4[d]	5.0±3.5[a]	2.2±2.4[e]	3.8±2.0[abc]
24:1n-9	0.6±0.1[a]	0.6±0.2[a]	0.6±0.2[a]	0.6±0.1[ab]	0.5±0.2[cd]	0.5±0.2[bc]	0.5±0.1[bcd]	0.5±0.2[bc]	0.4±0.1[e]	0.5±0.1[d]
Total Monoenes[2]	***31.5±5.4[a]***	***30.9±4.6[a]***	***36.0±7.3[b]***	***35.0±3.7[bc]***	***33.7±5.1[c]***	***38.3±6.0[d]***	***39.0±6.4[d]***	***44.6±4.2[e]***	***44.7±3.8[e]***	***45.5±3.8[e]***
18:2n-6	5.2±0.7[abc]	5.2±1.1[ab]	4.8±1.7[a]	4.6±1.7[a]	5.8±1.4[bc]	6.0±2.3[c]	7.7±2.5[d]	8.7±3.1[e]	11.1±2.4[f]	10.3±2.0[g]
20:2n-6	0.4±0.0[a]	0.4±0.1[a]	0.4±0.1[a]	0.3±0.1[a]	0.4±0.1[a]	0.5±0.2[b]	0.5±0.1[c]	0.6±0.2[d]	0.7±0.2[e]	0.7±0.2[f]
20:4n-6	0.8±0.1[ab]	0.9±0.1[a]	0.9±0.2[a]	0.8±1.1[ab]	0.8±0.1[bc]	0.7±0.2[cd]	0.7±0.2[d]	0.6±0.2[e]	0.5±0.1[f]	0.5±0.1[g]
Total n-6 PUFA[3]	***7.2±0.9[abc]***	***7.3±1.2[abc]***	***6.9±1.8[c]***	***6.7±1.8[bc]***	***7.6±1.6[ab]***	***7.8±2.2[a]***	***9.6±2.5[d]***	***10.3±3.2[e]***	***12.8±24[f]***	***12.0±2.1[g]***
18:3n-3	1.0±0.2[ab]	0.8±0.2[a]	1.2±0.6[bc]	1.3±0.3[c]	1.2±0.3[bc]	1.8±0.8[d]	2.2±1.2[e]	2.8±1.2[f]	3.7±0.8[g]	3.7±0.9[g]
18:4n-3	1.8±0.2[a]	1.7±0.2[ab]	1.6±0.2[c]	1.6±0.2[bc]	1.8±0.2[a]	1.6±0.4[c]	1.3±0.3[d]	1.2±0.3[e]	1.0±0.3[f]	1.1±0.3[g]
20:4n-3	1.4±0.1[a]	1.4±0.1[a]	1.4±0.2[a]	1.4±0.2[a]	1.4±0.1[a]	1.4±0.3[a]	1.2±0.3[b]	1.1±0.2[c]	1.0±0.2[d]	1.1±0.2[c]
20:5n-3 (EPA)	12.2±2.6[ab]	12.4±2.0[a]	11.4±3.2[bc]	10.8±1.6[c]	10.8±2.2[c]	9.2±2.6[d]	8.1±2.3[e]	6.2±1.4[f]	5.9±1.5[g]	5.5±1.5[g]
22:5n-3 (DPA)	4.2±0.8[a]	4.5±0.7[b]	4.5±1.0[b]	4.4±0.6[ab]	4.0±0.8[a]	3.6±1.0[c]	3.3±0.9[d]	2.5±0.5[e]	2.3±0.5[f]	2.3±0.6[f]
22:6n-3 (DHA)	12.3±0.9[a]	12.1±1.6[a]	10.5±1.1[b]	10.4±1.6[bc]	10.9±1.1[b]	9.9±2.0[c]	8.7±2.3[d]	8.1±1.7[e]	7.6±1.3[f]	7.4±1.5[f]
Total n-3 PUFA[4]	***32.9±3.6[a]***	***33.0±2.4[a]***	***30.6±3.8[b]***	***30.1±2.2[b]***	***30.3±2.7[b]***	***27.7±4.2[c]***	***25.0±4.7[d]***	***22.0±2.7[e]***	***21.7±2.6[e]***	***21.5±2.7[e]***
Total PUFA[5]	***42.6±4.9[a]***	***42.9±3.5[a]***	***39.7±5.2[b]***	***39.1±3.2[b]***	***40.3±4.1[b]***	***37.5±4.2[c]***	***36.4±4.5[d]***	***33.6±3.4[e]***	***35.8±2.7[d]***	***34.8±2.3[f]***
n-3:n-6	4.6±0.6[ab]	4.5±0.8[a]	4.4±1.2[a]	4.5±1.2[a]	4.0±1.0[bc]	3.6±1.4[c]	2.6±1.2[d]	2.1±0.9[e]	1.7±0.7[f]	1.8±0.5[f]
EPA ($g.100g^{-1}$)	1.4±0.5[a]	1.4±0.4[a]	1.1±0.5[b]	1.2±0.5[b]	1.1±0.4[b]	0.9±0.3[c]	0.9±0.3[c]	0.7±0.3[d]	0.7±0.3[d]	0.6±0.2[e]
DPA ($g.100g^{-1}$)	0.5±0.2[a]	0.5±0.2[b]	0.5±0.2[ac]	0.5±0.2[ac]	0.4±0.2[c]	0.4±0.1[d]	0.4±0.1[d]	0.3±0.1[e]	0.3±0.1[e]	0.2±0.1[e]
DHA ($g.100g^{-1}$)	1.4±0.3[a]	1.4±0.3[a]	1.0±0.3[bc]	1.1±0.3[bc]	1.1±0.3[b]	1.0±0.2[cd]	0.9±0.3[d]	0.8±0.2[e]	0.9±0.2[e]	0.8±0.2[f]
EPA+DHA ($g.100g^{-1}$)	2.7±0.8[a]	2.8±0.7[a]	2.2±0.7[b]	2.2±0.8[b]	2.2±0.7[b]	1.9±0.5[c]	1.8±0.6[c]	1.5±0.5[d]	1.5±0.5[d]	1.4±0.4[e]
EPA+DPA+DHA ($g.100g^{-1}$)	3.2±1.0[a]	3.3±0.8[a]	2.6±0.9[b]	2.7±1.0[b]	2.6±0.9[b]	2.3±0.6[c]	2.2±0.7[c]	1.7±0.6[d]	1.8±0.5[d]	1.6±0.5[e]

[1]includes 15:0, 22:0 and 24:0
[2]includes 16:1n-9, 20:1n-11, 20:1n-7 and 22:1n-9
[3]includes 18:3n-6, 20:3n-6, 22:4n-6 and 22:5n-6
[4]includes 20:3n-3 and 21:5n-3
[5]includes 16:2, 16:3 and 16:4
